# Contagiousness of Scarlatina

**Published:** 1855-09

**Authors:** Wm. G. Meecham

**Affiliations:** Warsaw, Wyoming Co., N. Y.


ART. III.— *Contagiousness of Scarlatina.* By WM. G. MEECHAM, M. D., Warsaw, Wyoming Co., N. Y.

Having recently come into possession of a legacy of facts rich in scientific interest, in that they present (to my judgment at least) indubitable proof of the contagiousness of scarlatina, a doctrine which, though generally to be found in the creeds of physicians of the nineteenth century, is yet ignored by some, and, in not a few instances, those too of men of no mean capacity and attainment, I will now transmit that legacy to you in a check upon the bank of epistolary correspondence, with a request that, if deemed of sufficient worth, it be distributed among your monthly readers in a check on the bank of the Buffalo Medical Journal.

This contagiousness having been doubted by no less a man than Dr. Dewees, who has stated that he had never seen "any decided proof that it (scarlatina) had communicated itself in any one instance," I certainly think I cannot justly be accused of engaging in supererogatory labor in endeavoring, by making reasonable deductions from actual occurrences under my own observation, to corroborate the truth of the doctrine.

The facts I allude to are substantially as follows: Scarlatina, under each of its three varieties of phase, as simplex, anginosa and maligna, has been prevailing, to a limited extent, in our own town and in one or more of those adjoining, during the last eight or twel e weeks. My partner and myselt have treated several of the cases, and among them an unusually severe one of the malignant form, which, thanks to the successful agency of therapeutic management and the recuperative energy of nature, has been mastered, and the patient is now rapidly advancing in the highway of convalescence. At my daily visits to this patient I frequently remained in her chamber from thirty to sixty minutes, and a large share of this time in close proximity to her person, either conducting the ordinary examination or removing and reapplying dressings and bandages from and to ulcers and superficial abscesses with which she was most bountifully supplied, and which incessantly discharged inordinate quantities of a sero-purulent fluid. By this means my *clothing* doubtless became thoroughly impregnated with the effluvium from her person, and would it be making too large a draft upon enlightened reason to say my *body* also? During my daily attendance upon this patient, it so happened that I slept for three successive nights with a son of my partner, a boy nine years of age. On the day succeeding the third night the boy manifested well-marked prodromata of scarlatina, such as languor, rigors alternating with flushes of heat, a frequent pulse, hot dry skin, furred tongue, anorexia, thirst, muscular weakness, stupor, &c. The next day the peculiar rash presented itself, gradually augmenting in intensity and extent, until well nigh the entire surface of the body was tinged with the characteristic "boiled lobster" hue. The disease pursued the steady, straightforward course of the simplex variety, and upon the fifth day succeeding the first appearance of the efflorescence, reached its acme and began to descend the declivity of resolution. Upon the same day (the fifth) a sister of the boy, aged five years, returned home, having been absent in Wisconsin four weeks, and on the third day thereafter was in turn attacked by the same disease, but of a severer form, the anginosa. The boy, be it remembered, had been subjected to the epidemic influences, if such existed, for a period of many weeks previously, but they had not proved sufficient to institute the malady in his person. It was not until I slept with him, after having been daily in the chamber of the lady affected with the malignant type of scarlatina, that the boy's system was contaminated.

Now let me ask, what are the inferences logically and justly deducible from these premises? Is not one of them this, that the scarlatina of the boy was induced by a specific contagion? Else, why should the boy so long have escaped, and been seized at that particular time? Some may be ready here to put in the plea, "coincidence;" but no reason, no philosophy requires such a plea. The *prima facie* evidence of the existence of the relationship of cause and effect between these two circumstances, is, to say the least, extremely weighty. Is it not proper to infer from this correlation of incidents that my apparel became laden with a peculiar subtile effluvium from the skin and bronchio-pulmonary mucous membrane of my patient, or even that my very body was more or less contaminated with the infinitesimal particles of such a specific poison, and that the boy, from close proximity to the fomites and my own person, was brought within the range of a contagious influence? My own judgment points an unwavering finger to this inference.

It may be asked, if there was contagion, why had I not been the vehicle of its conveyance to the boy long anteriorly, since I had been in attendance upon scarlatina patients for a considerable time previously? For three reasons, I reply:

First, because the cases that I had treated before had been of the milder varieties, and the contagion consequently less concentrated and virulent.

Secondly, because I had not been necessitated to spend as much time over their persons, and thus allow of opportunity for the fixation of the contagious miasm in my dress and person.

And lastly, because I had not brought the boy into continued, immediate proximity to my own infected body and apparel, as I afterwards did by sleeping with him.

And here, by way of digression, assuming for the nonce the doctrine of contagion proved, let me say a word in relation to the idea just advanced, that the body of an individual, a physician or a nurse for instance, who has been protected against the peculiar agency of scarlatina by an antecedent attack, may become freighted with the invisible contagion, at least upon its superficies, without suffering its morbific effects, so as to be able to communicate it to those unprotected. The case now under consideration gives evidence to the point—The boy did not suffer from the disease *until after he had slept with me.* I had had scarlet fever in boyhood, and just then furnished not one symptom of an incipient second attack, nor have I since. Indeed, I see nothing unreasonable in the hypothesis. One great characteristic of eruptive fevers, it is well known, is their occurrence, in the main, but once in the life of an individual. The invisible workings of the virus seem to impress upon the system such changes as are ever after prophylactic against the same agent. What this change precisely is, whether, according to Liebig, a process of fermentation, or, according to others, something else, it is of little moment to our present purpose to stop to consider. Suffice it to say that in view of the acknowledged protective virtue of an assault of the disease, I see nothing in the way of the philosophy of the theory indicated above. May not the contagious miasma be fixed upon the cuticle of a protected person, aye, may it not even enter his circulation, without occasioning the disease? I think no one need hesitate long to reply in the affirmative. It is unreasonable to suppose that scarlatina renders the integument and gastro-pulmonary mucous membrane ever after impervious to its peculiar poison, but open and free to every other noxious virus.

Again; to resume the thread of our argument, the girl's case presents nearly as valuable testimony in favor of contagion as does that of the boy. Previously to going West, she had been subject to the epidemic influences for at least six weeks, and had escaped unscathed. During her stay in Wisconsin, she had had no symptoms of the disease in question. But almost immediately after her return and exposure to the disease of her brother, (three days,) she evinces the rage of the "scarlet" monster. If the cause were merely epidemic, why had she not been assailed prior to that time, and not so directly after she had been brought in proximity to the boy? Assuredly the nose is very prominent on this face.

We have also in these facts evidence corroborative of certain statements founded on the sure basis of accumulated experience, viz., first, that the poisons of the various forms of the fever is virulent proportionably with the severity of the variety, making due allowance for the qualifying circumstances, such as constitution, age, time, place, &c.; and secondly, that one grade of the disease may induce in a different individual another. The scarlatina maligna of the lady occasioned the scarlatina simplex of the boy, while the scarlatina simplex of the boy instituted the scarlatina anginosa of the girl. Hence it is inferable that the three forms are one and the same affection, modified by prevailing epidemic influences, temperament, &c.

The true doctrine in relation to the etiology of scarlatina, as established by accumulated experiences, and confirmed by analogy, is doubtless this:—In the vast majority of instances epidemic and contagious influences conjoin in the causation of this (in its sequelæ) protean malady; and yet there is no valid ground for disbelieving the possibility of its generation, *de novo*, in the appropriate combination of circumstances. But to me it seems inexplicable that so intelligent and practical a physician as Dr. Dewees, should for a moment entertain doubt respecting the existence of the contagiousness of scarlatina. But I cease to wonder. *The immortal Shakspeare poached!*

Another circumstance attending this epidemic, perchance worthy of a passing notice, is its occurrence at and near the vernal equinox, an incident corroborative of the statement of several authors that scarlatina prevails more frequently about the equinoxial seasons than at any other time.

Warsaw, Wyoming Co., July 9, 1855.

